# Association between metabolic disorders and clinicopathologic features in endometrial cancer

**DOI:** 10.3389/fendo.2024.1351982

**Published:** 2024-08-26

**Authors:** Yuanpei Wang, Qianwen Liu, Yi Sun, Weijia Wu, Xiaoran Cheng, Xuerou Chen, Fang Ren

**Affiliations:** Department of Obstetrics and Gynecology, The First Affiliated Hospital of Zhengzhou University, Hangzhou, China

**Keywords:** endometrial cancer, hyperglycemia, hypertension, obesity, lymph node metastasis

## Abstract

**Background:**

In recent years, the incidence of Endometrial cancer (EC) has been on the rise due to high-fat, high-calorie diets and low-exercise lifestyles. However, the relationships between metabolic disorders and the progression of EC remain uncertain. The purpose of our study was to explore the potential association between obesity, hypertension, hyperglycemia and clinicopathologic characteristics in EC patients.

**Methods:**

In categorical variables, Chi-square tests were used to calculate *P* values. Univariate logistic regression and multivariate logistic regression were used to identify the risk factors of myometrial invasion>1/2 and lymph node metastasis. Overall survival (OS) was estimated using the Kaplan-Meier method.

**Results:**

The study included 406 individuals with EC, 62.6% had type I and 37.4% had type II. Hypertension was seen in 132 (32.5%), hyperglycemia in 75 (18.5%), and overweight or obesity in 217 (53.4%). Hypertension, hyperglycemia, and obesity are strongly associated with the clinicopathologic features of EC. Multivariate logistic regression revealed that hyperglycemia (OR=2.439,95% CI: 1.025-5.804, *P =* 0.044) was a risk factor for myometrial invasion depth >1/2 in patients with type I EC, and hypertension (OR=32.124,95% CI: 3.287-313.992, *P =* 0.003) was a risk factor for lymph node metastasis in patients with type I EC. Survival analysis found that hyperglycemia (*P* < 0.001) and hypertension (*P* = 0.002) were associated with OS in type I EC. Neither hyperglycemia, hypertension, nor obesity were associated with the prognosis in type II EC.

**Conclusion:**

Hyperglycemia was a risk factor for myometrial invasion depth >1/2 in patients with type I EC and hypertension was a risk factor for lymph node metastasis in patients with type I EC. Hypertension and hyperglycemia were associated with poor prognosis in patients with type I EC.

## 1 Introduction

EC is one of the three major malignant tumors of the female reproductive system, and the incidence of EC has been on the rise due to high-fat, high-calorie diets and low-exercise lifestyles in recent years (1). It is estimated that there are about 66,200 new cases and 13,030 deaths in 2023 (2). EC can be classified into type I and type II based on clinical and molecular features (3). Type I EC is associated with excessive estrogen exposure and hormone receptor-positive status, which account for approximately 80% of all EC cases, and usually have favorable prognosis. Type II EC is primarily characterized by overexpression of estrogen and progesterone receptors (4, 5). The risks associated with EC include menarche early, menopause late, nulliparity, hormone therapy after menopause, and obesity (6).

Metabolic disorders play an important role in the development and progression of EC (7–10). Previous research has shown that women with metabolic disorders have a higher risk of EC and are also associated with a poor prognosis. Obesity is recognized as a significant risk factor for EC (11–14). In symptomatic premenopausal women, those with a BMI exceeding 25, 30, and 40 had a 3.85, 5.25, and 19.79 times greater likelihood of developing EC compared to women with a normal BMI, respectively (15). Furthermore, obese EC patients exhibit diminished responsiveness to standard treatment, an increased occurrence of distant metastases, and a poorer prognosis (16, 17). Hyperglycemia and hypertension are also essential risk factors for the development of EC and are closely related to the clinicopathological features and prognosis of EC. A meta-study revealed that diabetes was associated with an increased risk of EC (18).

However, the current understanding of the relationships between metabolic disorders and the progression of EC remains uncertain. Consequently, we performed a retrospective study to explore the potential association between obesity, hypertension, hyperglycemia and clinicopathologic characteristics in EC patients.

## 2 Methods

### 2.1 Study population

EC patients from the medical record system of the First Affiliated Hospital of Zhengzhou University (from January 1, 2012, to December 31, 2020) were included in this study. This study was approved by the Ethics Committee of the First Affiliated Hospital of Zhengzhou University (Ethics Approval Number: 2023-KY-0305-002). Criteria for inclusion were as follows: (1) EC diagnosed by the First Affiliated Hospital of Zhengzhou University after staged surgery; (2) No neoadjuvant before surgery. Exclusion criteria:(1) Patients with other malignant tumors; (2) Patients not undergoing surgery; (3) Patients with preserved fertility; (4) Patients with incomplete case information.

### 2.2 Clinical data collection

Patient information was collected through the case system of the First Affiliated Hospital of Zhengzhou University as follows: age at the time of diagnosis, height, weight, blood pressure, blood glucose, date of diagnosis, surgical stage, type of pathology, pathologic grade, lymph node metastasis, parametrial invasion, stromal invasion, myometrial invasion and survival time. OS was defined as the date from the date of diagnosis to the date of death from any cause or final follow-up. BMI was classified as not overweight (BMI<25) or overweight and obesity (BMI≥25). Hyperglycemia was defined as a situation of fasting glucose>6.1 mmol/L or 2-hour glucose>7.8. Hypertension was defined by casual blood pressure >140/90 mmHg. Surgical staging according to the 2009 International Federation of Gynecology and Obstetrics (FIGO) staging criteria for EC.

### 2.3 Statistical analysis

The continuous variables that conform to normal distribution were expressed as mean ± SD and those that did not were expressed as median (interquartile range). In categorical variables, Chi-square tests and Fisher's exact test are used to calculate *P-*values. Univariate logistic regression and multivariate logistic regression were used to identify the risk factors of myometrial invasion>1/2 and lymph node metastasis. In the multivariable logistic regression analysis, only factors with a *P* value less than 0.1 were included. The results were expressed as odds ratio (OR). OS was estimated using the Kaplan-Meier method, and the *P*-values were compared using the log-rank test. All statistical analyses were performed with SPSS26. *P <* 0.05 was considered statistically significant.

## 3 Results

### 3.1 Baseline characteristics of the study population

A cohort of 406 individual samples diagnosed with EC was included in our study. Of these, 254 (62.6%) had type I EC, while 152 (37.4%) had type II EC. Hypertension was present in 132 individuals (32.5%), hyperglycemia in 75 individuals (18.5%), and obesity in 217 individuals (53.4%). Out of 132 hypertensive patients, 114 took daily medication to control their blood pressure. Among 75 patients with hyperglycemia, 69 took daily medication to control their blood sugar levels.The distribution of cancer stages among the patients was as follows: 312 (76.8%) were classified as stage I, 17 (4.2%) as stage II, 61 (15.0%) as stage III, and 11 (2.7%) as stage IV. Lymph node metastasis was detected in 60 patients (14.7%), while 118 patients (29%) exhibited myometrial invasion > 1/2. Additionally, 13 patients (3.2%) displayed cervical stromal invasion (**Table 1**).

**Table 1:** Baseline characteristics for 406 EC patients.

Characteristic	Number of patients (%)
Age(years)	
<55	171(42.1)
≥55	235(57.9)
Menopause	
No	112(27.6)
Yes	294(72.4)
Hyperglycemia	
Negative	331(81.5)
Positive	75(18.5)
Hypertension	
Negative	274(67.5)
Positive	132(32.5)
Overweight or obese	
Negative	189(46.6)
Positive	217(53.4)
Pathologic type	
I	254(62.6)
II	152(37.4)
Histologic grading	
G1+G2	223(54.9)
G3	74(18.2)
Unknown	109(26.8)
Myometrial invasion	
≤1/2	288(70.9)
>1/2	118(29.1)
Cervical stromal invasion	
Negative	393(96.8)
Positive	13(3.2)
Parametrial invasion	
Negative	402(99.0)
Positive	4(1.0)
Lymph node metastasis	
Negative	346(85.2)
Positive	60(14.8)
Staging	
1	312(76.8)
2	17(4.2)
3	61(15.0)
4	11(2.7)
Unknown	5(1.2)

### 3.2 Correlation of hypertension, hyperglycemia, and obesity with EC clinical parameters

In patients with type I EC, hyperglycemia was associated with age (*P* < 0.001), menopause (*P*=0.002), and myometrial invasion depth >1/2 (*P* = 0.011), whereas hyperglycemia was not associated with these factors in patients with type II EC. Hypertension was correlated with age (older than 55 years, *P* = 0.002), menopause (*P* = 0.008), myometrial invasion depth >1/2 (*P* = 0.031), lymph node metastasis (*P* < 0.001), and FIGO stage (*P* = 0.014) in type I EC. In type II EC, hypertension is associated with advanced age (older than 55 years, *P* = 0.01), menopause (*P* = 0.001), pathologic grade(*P*=0.034), and FIGO stage (*P* = 0.013). Obesity was correlated with cervical stromal invasion in both type I (*P* = 0.022) and type II (*P* = 0.019) EC (**Table 2**).

**Table 2:** Correlation of hypertension, hyperglycemia, and obesity with EC clinicopathology.

Variable	Type I							Type II						
	Patients	Hyperglycemia		Hypertension		Overweight or obese		Patients	Hyperglycemia		Hypertension		Overweight or obese	
		N (%)	*P* value	N (%)	*P* value	N (%)	*P* value		N (%)	*P* value	N (%)	*P* value	N (%)	*P* value
Age(years)			**<0.001**		**0.002**		0.253			0.068		**0.010**		0.126
<55	127	14(11.0)		29(22.8)		69(54.3)		44	3(6.8)		8(18.2)		16(36.4)	
≥55	127	38(29.9)		52(40.9)		78(61.4)		108	20(18.5)		43(39.8)		54(50.0)	
Menopause			**0.002**		**0.008**		0.502			0.497		**0.003**		0.209
No	96	10(10.4)		21(21.9)		53(55.2)		16	1(6.25)		0(0)		5(31.25)	
Yes	158	42(26.6)		60(38.0)		94(59.5)		136	22(16.2)		51(0.375)		65(47.8)	
Histologic grading			0.769		1.000		0.462			1.000		**0.034**		0.118
G1+G2	209	45(21.5)		66(31.6)		125(59.8)		14	1(7.1)		1(7.1)		3(21.4)	
G3	19	3(15.8)		6(31.6)		13(68.4)		55	4(7.3)		23(41.8)		27(49.1)	
Myometrial invasion			**0.011**		**0.031**		0.298			0.533		0.380		0.143
≤1/2	191	32(16.8)		54(28.3)		107(56.0)		97	16(16.5)		35(36.1)		49(50.5)	
>1/2	63	20(31.7)		27(42.9)		40(63.5)		55	7(12.7)		16(29.1)		21(38.2)	
Cervical stromal invasion			0.311		0.418		**0.022**			0.345		0.864		**0.019**
Negative	246	52(21.1)		81(32.9)		139(56.5)		147	21(14.3)		50(34.0)		65(44.2)	
Positive	8	0(0)		1(12.5)		8(100.0)		5	2(40.0)		1(20.0)		5(100.0)	
Parametrial invasion			1.000		1.000		0.421			0.940		1.000		1.000
Negative	253	52(20.6)		81(32.0)		147(58.1)		149	22(14.8)		50(33.6)		69(46.3)	
Positive	1	0(0)		0(0)		0(0)		3	1(33.3)		1(33.3)		1(33.3)	
Lymph node metastasis			0.267		**<0.001**		0.177			0.812		0.099		0.586
Negative	230	45(19.6)		63(27.4)		130(56.5)		116	18(15.5)		43(37.1)		52(44.8)	
Positive	24	7(29.2)		18(75.0)		17(70.8)		36	5(13.9)		8(22.2)		18(50.0)	
Staging			0.103		**0.014**		0.193			0.506		**0.013**		0.835
1	211	41(19.4)		61(28.9)		123(58.3)		101	16(15.8)		41(40.6)		46(45.5)	
2	11	6(54.5)		7(63.6)		9(81.8)		6	0(0)		0(0)		3(50.0)	
3	25	5(20.0)		9(36.0)		11(44.0)		36	7(19.4)		9(25.0)		18(50.0)	
4	2	0(0)		2(100.0)		1(50.0)		9	0(0)		1(11.1)		3(33.3)	

The bold numbers in the table indicate a statistically significant difference in the P-values.

### 3.3 Pathologic risk factors for EC: a univariate analysis

Next, we analyzed the risk factors for lymph node metastasis and myometrial invasion >1/2 for type I and type II EC, respectively, using univariate logistic analysis. These results revealed that the risk factors for lymph node metastasis in type I EC were as follows: myometrial infiltration >1/2(OR= 9.714, 95% CI: 3.804-24.809, *P* < 0.001), hypertension (OR = 7.952, 95% CI:3.020-20.943, *P* < 0.001), stage (*P* < 0.001), histologic grading (OR = 1.879, 95% CI: 1.187-2.973, *P* = 0.007) (**Table 3**). Risk factors for type I EC with myometrial invasion >1/2 were age (OR = 1.902, 95% CI: 1.062-3.406, *P* = 0.031), lymph node metastasis (OR = 9.714, 95% CI: 3.804-24.809, *P* < 0.001), cervical stromal invasion(OR = 9.947, 95% CI: 1.954-50.642, *P* = 0.006), hypertension (OR=1.903, 95%CI:1.055-3.433, *P*=0.033), hyperglycemia (OR=2.311, 95%CI:1.204-4.438, *P*=0.012 ), staging (*P* < 0.001), and histologic grading (OR = 2.507, 95%CI: 0.953-6.592, *P*=0.062) (**Table 3**). These results found that the risk factors for lymph node metastasis of type II EC were: menopause (OR = 3.857, 95%CI: 1.330-11.183, *P*=0.013), myometrial invasion > 1/2 (OR = 3.376, 95%CI: 1.557-7.321, *P*=0.002), cervical stromal invasion (OR = 14.375, 95%CI: 1.552-133.158, *P*=0.019), and histologic grading (OR = 3.375, 95%CI: 0.956-11.909, *P*=0.059) (**Table 4**). Risk factors for type II EC with myometrial infiltration >1/2 were: lymph node metastasis (OR = 3.376, 95%CI: 1.557-7.321, *P*=0.002), cervical stromal invasion (OR = 2.323, 95%CI: 0.253-21.317, *P*=0.006), stage (*P*=0.014), and histologic grading (OR = 8.348, 95%CI: 1.702-40.933, *P*=0.009) (**Table 4**).

**Table 3:** Univariate regression analysis of risk factors associated with lymph node metastasis and myometrial invasion >1/2 in type I EC.

Variable	Lymph node metastasis			Myometrial invasion>1/2		
	OR	95%CI	*P* value	OR	95%CI	*P* value
Age	1.756	(0.739-4.174)	0.203	1.902	(1.062-3.406)	**0.031**
Menopause	1.239	(0.509-3.016)	0.636	1.421	(0.776-2.601)	0.255
Myometrial invasion>1/2	9.714	(3.804-24.809)	**<0.001**	NA	NA	NA
Lymph node metastasis	NA	NA	NA	9.714	(3.804-24.809)	**<0.001**
Cervical stromal invasion	3.394	(0.646-17.834)	0.149	9.947	(1.954-50.642)	**0.006**
Parametrial invasion	NA	NA	1.00	0.000	(0.000-0.000)	1.000
Hypertension	7.952	(3.020-20.943)	**<0.001**	1.903	(1.055-3.433)	**0.033**
Hyperglycemia	1.693	(0.662-4.327)	0.272	2.311	(1.204-4.438)	**0.012**
Overweight or obese	1.868	(0.746-4.678)	0.182	1.365	(0.759-2.456)	0.299
Staging			**<0.001**			**<0.001**
1	Ref	Ref	Ref	Ref	Ref	Ref
2	43.125	(9.198-202.197)	**<0.001**	14.917	(3.756-59.244)	**<0.001**
3	65.864	(18.572-233.574)	**<0.001**	9.944	(4.047-24.438)	**<0.001**
4	51.750	(2.727-981.935)	**<0.001**	9036562402	(0.000-0.000)	0.999
Histologic grading	1.879	(1.187-2.973)	**0.007**	2.507	(0.953-6.592)	**0.062**

OR, odds ratio; CI, confidence interval; NA, Not applicable, Not included in regression models. The bold numbers in the table indicate a statistically significant difference in the P-values.

**Table 4:** Univariate regression analysis of risk factors associated with lymph node metastasis and myometrial invasion >1/2 in type II EC.

Variable	Lymph node metastasis			Myometrial invasion>1/2		
	OR	95%CI	*P* value	OR	95%CI	*P* value
Age	1.824	(0.829-4.013)	0.135	1.311	(0.623-2.759)	0.475
Menopause	3.857	(1.330-11.183)	**0.013**	1.894	(0.668-5.367)	0.230
Myometrial invasion>1/2	3.376	(1.557-7.321)	**0.002**	NA	NA	NA
Lymph node metastasis	NA	NA	NA	3.376	(1.557-7.321)	**0.002**
Cervical stromal invasion	14.375	(1.552-133.158)	**0.019**	2.323	(0.253-21.317)	**0.006**
Parametrial invasion	5678638842	(0.000-0.000)	0.999	3.623	(0.321-40.894)	0.298
Hypertension	0.485	(0.203-1.159)	0.104	0.727	(0.356-1.485)	0.381
Hyperglycemia	0.878	(0.301-2.560)	0.812	0.738	(0.283-1.923)	0.534
Overweight or obese	1.231	(0.582-2.602)	0.587	0.605	(0.308-1.187)	0.144
Staging			0.753			**0.014**
1	Ref	Ref	Ref	Ref	Ref	Ref
2	1	NA	1	2.607	(0.496-13.692)	0.257
3	NA	NA	0.995	2.333	1.063-5.120	**0.035**
4	NA	NA	0.996	9.125	(1.787-46.608)	**0.008**
Histologic grading	3.375	(0.956-11.909)	**0.059**	8.348	(1.702-40.933)	**0.009**

OR, odds ratio; CI, confidence interval; NA, Not applicable, Not included in regression models. The bold numbers in the table indicate a statistically significant difference in the P-values.

### 3.4 Pathologic risk factors for EC: a multivariate analysis

Multivariate logistic regression revealed that the factors for lymph node metastasis in type I EC were: hypertension (OR = 32.124,95% CI: 3.287-313.992, *P* = 0.003) and stage (*P* = 0.002) (**Table 5**). The risk factors for myometrial invasion >1/2 in type I EC were: lymph node metastasis (OR = 5.472, 95% CI: 1.014-29.515, *P* = 0.048), cervical stromal invasion (OR = 10.231, 95% CI: 1.353-77.373, *P* = 0.024), hyperglycemia (OR=2.439, 95% CI: 1.025-5.804, *P* = 0.044), and stage (*P* = 0.044) (**Table 5**). The risk factor for myometrial invasion >1/2 in type II EC was related to histologic grading (OR = 2.798, 95% CI: 0.836-9.364, *P* = 0.016) (**Table 6**).

**Table 5:** Multivariate regression analysis of risk factors associated with lymph node metastasis and myometrial invasion >1/2 in type I EC.

Variable	Lymph node metastasis			Myometrial invasion>1/2		
	OR	95%CI	*P* value	OR	95%CI	*P* value
Age	NA	NA	NA	1.502	(0.657-3.437)	0.335
Myometrial invasion >1/2	3.749	(0.592-23.744)	0.161	NA	NA	NA
Lymph node metastasis	NA	NA		5.472	(1.014-29.515)	**0.048**
Cervical stromal invasion	NA	NA	NA	10.231	(1.353-77.373)	**0.024**
Hypertension	32.124	(3.287-313.992)	**0.003**	1.205	(0.504-2.882)	0.674
Hyperglycemia	NA	NA	NA	2.439	(1.025-5.804)	**0.044**
Staging			**0.002**			**0.044**
1	Ref	Ref	Ref	Ref	Ref	Ref
2	105.914	(315.161-3124.318)	<0.001	2.902	(0.426-19.777)	0.276
3	379.867	(44.161-13280.700)	<0.001	8.337	(2.178-31.921)	0.002
4	NA	NA	1	NA	NA	NA
Histologic grading	11.965	(0.805-177.896)	0.072	2.442	(0. 705-8.452)	0.159

OR, odds ratio; CI, confidence interval; NA, Not applicable, Not included in regression models. The bold numbers in the table indicate a statistically significant difference in the P-values.

**Table 6:** Multivariate regression analysis of risk factors associated with lymph node metastasis and myometrial invasion >1/2 in type II EC.

Variable	Lymph node metastasis			Myometrial invasion>1/2		
	OR	95%CI	*P* value	OR	95%CI	*P* value
Menopausal	3.761	(0.857-16.496)	0.079	NA	NA	NA
Myometrial invasion >1/2	2.820	(0.730-10.899)	0.133	NA	NA	NA
Cervical stromal invasion	0.000	NA	1.000	212482974.0	NA	1
Staging						0.720
1	NA	NA	NA	Ref	Ref	Ref
2	NA	NA	NA	6.228	(1.273-30.465)	0.751
3	NA	NA	NA	17.041	(5.455-53.235)	0.294
4	NA	NA	NA	13932071401	NA	0.737
Histologic grading	1.557	(0.368-6.586)	0.547	2.798	(0.836-9.364)	**0.016**

OR, odds ratio; CI, confidence interval; NA, Not applicable, Not included in regression models.

### 3.5 Relationship between hypertension, hyperglycemia, obesity, and prognosis of patients with EC

**Figure 1:**
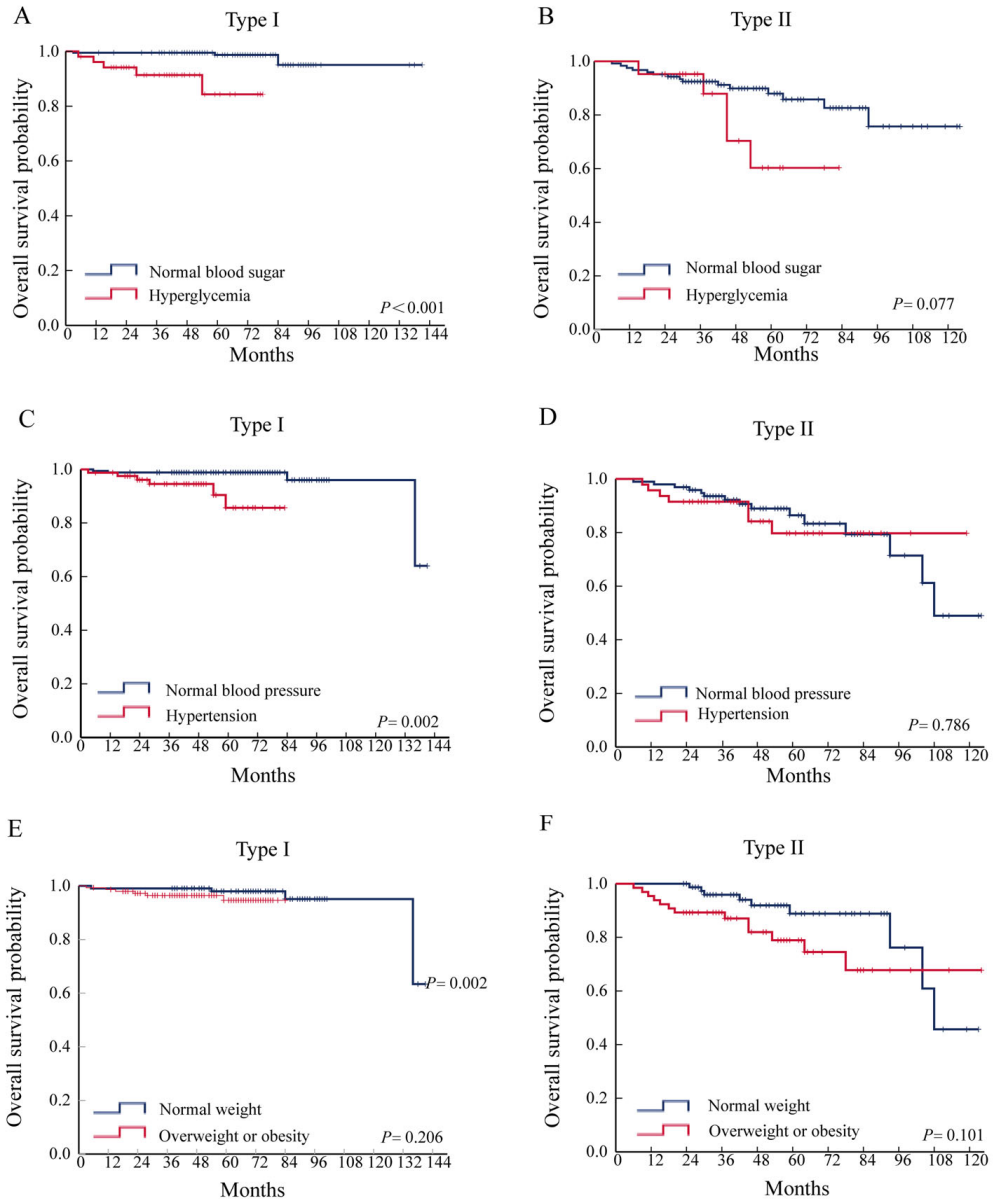
Relationship between hypertension, hyperglycemia, obesity, and prognosis of patients with EC. **(A)** Effect of hyperglycemia on OS in type I EC. **(B)** Effect of hyperglycemia on OS in type II EC. **(C)** Effect of hypertension on OS in type I EC. **(D)** Effect of hypertension on OS in type II EC. **(E)** Effect of overweight or obesity on OS in type I EC. **(F)** Effect of overweight or obesity on OS in type II EC.

Survival analysis found that hyperglycemia (*P* < 0.001) and hypertension (*P* = 0.002) were associated with OS in type I EC. Neither hyperglycemia, hypertension, nor obesity were associated with the prognosis of type II EC (**Figure 1**).

## 4 Discussion

EC is one of the cancers most closely associated with metabolic disease, obesity, hypertension, and diabetes, which are known as the metabolic triad of EC (3). This study identified hypertension as a significant risk factor for lymph node metastasis in type I EC. Additionally, hypertension and hyperglycemia were found to be risk factors for deep myometrial invasion and were further associated with an unfavorable prognosis in type I EC.

Hyperglycemia is closely associated with the development and progression of many malignant tumors such as ovarian, pancreatic, and breast cancer (19, 20). A large number of epidemiologic studies on the subject have shown hyperglycemia was a risk factor for the development of EC (9, 11, 21–24). A meta-analysis that included 17 prospective studies and 12 retrospective studies found diabetes mellitus was a risk factor for EC. Another study showed a significant 15% increased risk of cancer-specific death in EC patients with preexisting diabetes compared to patients without diabetes (25). Our research has shown in type I EC patients, hyperglycemia was associated with myometrial invasion depth >1/2 and poor prognosis. A significant proportion, approximately 93%, of Type I EC, specifically the endometrioid type, exhibit an absence of the phosphatase and tensin homolog (PTEN) or harbor mutations within the PI3K/Akt/mTOR pathways, which are associated with glucose metabolism, hyperglycemia stimulates changes in the above signaling pathways to increase the proliferation, invasion, and migration of tumor cells, so hyperglycemia is associated with deep myometrial invasion in patients with type I EC (26–28). For example, one study showed that EC cells (UECC, Ishikawa, RL95-2) cultured under high glucose conditions (25 mM) were more aggressive than physiological levels (5 mM) and had increased expression of EMT pathway-related proteins (29). We speculate that hyperglycemia is associated with deep myometrial invasion of type I EC but not with type II EC, which may be caused by different molecular characteristics, and there are no reports that hyperglycemia affects the prognosis and molecular characteristics of type II EC. We look forward to future research that will shed light and address this issue.

We also found hypertension to be a risk factor for lymph node metastasis in type I EC and was associated with a poor prognosis for EC. A few studies have been conducted on the association of hypertension with the clinicopathologic characteristics of EC (30–33). One retrospective study revealed that a relative risk of EC associated with hypertension of 1.6 (34). Results of a meta-analysis suggested a strong association between hypertension and EC (35). An observational study has shown that hypertension was positively associated with mortality from a variety of cancers, and our study is consistent with this finding that hypertension is associated with poor EC outcomes (36). Hypertension is closely related to the progression of other tumors, for example, hypertension is associated with the TNM stage of gastric cancer, and with the stage, tumor size, and lymph node metastasis of thyroid cancer, in this study we found that hypertension is associated with lymph node metastasis in type I EC (37, 38). Patients with hypertension often have disorders of renin-angiotensin system (RAS), and previous studies have shown that changes in the RAS system are closely related to the occurrence and development of EC (39). For example, angiotensin II has been shown to enhance the proliferative capacity of endometrial cancer cell lines (40). A study of 30 endometrial cancer samples showed that high expression of ATP6AP2, AGTR1, and ACE1 in the RAS was associated with the spread of endometrial cancer (41). Although our study found that hypertension is not related to lymph node metastasis and prognosis of type II EC, there are still some hypertensive patients in type II EC, and some antineoplastic drugs (anti-angiogenic drugs, bevacizumab, etc.) can cause hypertension, and we should individualize the treatment of hypertensive endometrial cancer patients.

It occurrences recognized that obesity is one of the most critical risk factors for development of type I EC, and a large number of studies have shown that obesity could also affect the prognosis of patients with EC. Studies have shown that obesity was associated with a two- to five-fold increased risk of EC in pre and post-menopausal women, Every 10-year increase of the adult overweight duration (BMI ≥ 25 kg/m^2^) has been reported to be correlated with 17% higher risk of postmenopausal EC (42, 43). Although a large quantity of studies have demonstrated that obesity is a risk factor for EC, the effect of obesity on the prognosis of EC is still controversial Gunderson's research found that BMI is associated with all-cause mortality, but not with EC-specific mortality (44). A meta-analysis showed that higher BMI (≥30 kg/m^2^) was associated with increased all-cause mortality (HR = 1.34, 95%CI = 1.12-1.59) and recurrence (HR = 1.28, 95%CI = 1.06-1.56) of EC (14). Another study that included 937 cases of EC showed no association between weight and OS (45). Our study found no significant difference in the prognosis of EC patients with normal weight (BMI <25) compared to those who were overweight or obese (BMI ≥25). Future prospective studies with large samples are still needed to further determine the effect of obesity on the prognosis of EC patients, to provide postoperative health guidance for EC patients and to improve their quality of life and prolong their living time.

## 5 Conclusion

Hyperglycemia was a risk factor for myometrial invasion depth >1/2 in patients with type I EC and hypertension was a risk factor for lymph node metastasis in patients with type I EC. Hypertension and hyperglycemia were associated with poor prognosis in patients with type I EC.

## Data Availability

The original contributions presented in the study are included in the article/supplementary material. Further inquiries can be directed to the corresponding author.
